# Shared Neural Mechanisms for the Prediction of Own and Partner Musical Sequences after Short-term Piano Duet Training

**DOI:** 10.3389/fnins.2017.00165

**Published:** 2017-04-04

**Authors:** Claudia Lappe, Sabine Bodeck, Markus Lappe, Christo Pantev

**Affiliations:** ^1^Department of Medicine, Institute for Biomagnetism and Biosignalanalysis, University of MünsterMünster, Germany; ^2^Department of Psychology, University of MünsterMünster, Germany

**Keywords:** mismatch negativity, musical training, prediction error, joint action, sensorimotor integration

## Abstract

Predictive mechanisms in the human brain can be investigated using markers for prediction violations like the mismatch negativity (MMN). Short-term piano training increases the MMN for melodic and rhythmic deviations in the training material. This increase occurs only when the material is actually played, not when it is only perceived through listening, suggesting that learning predictions about upcoming musical events are derived from motor involvement. However, music is often performed in concert with others. In this case, predictions about upcoming actions from a partner are a crucial part of the performance. In the present experiment, we use magnetoencephalography (MEG) to measure MMNs to deviations in one's own and a partner's musical material after both engaged in musical duet training. Event-related field (ERF) results revealed that the MMN increased significantly for own and partner material suggesting a neural representation of the partner's part in a duet situation. Source analysis using beamforming revealed common activations in auditory, inferior frontal, and parietal areas, similar to previous results for single players, but also a pronounced contribution from the cerebellum. In addition, activation of the precuneus and the medial frontal cortex was observed, presumably related to the need to distinguish between own and partner material.

## Introduction

Musical performance is in essence a social conduct. It is a prime model for the ability to cooperate and work together toward a common goal. A deeper understanding of the neurophysiological mechanisms in musical performance might therefore be acquired by looking at it from the view point of task-sharing neurophysiological experiments (D'Ausilio et al., 2015). Such experiments have suggested that own actions and the observation of other's actions are functionally similarly represented in the brain (Knoblich and Jordan, 2003; Sebanz et al., 2003; van Schie et al., 2004; Bosbach et al., 2005; de Bruijn et al., 2012).

Ensemble musicians need to work together to achieve a satisfying performance. To reach this, they need to have common knowledge about how the whole piece should sound. During joint performance, musicians may then anticipate the actions of other performers in order to facilitate synchronization, and predict not only their own sound, but also the sound of the other ensemble members or the musical duet partner (Keller, 2008). The musical material of the partner is indeed of considerable importance since it constitutes an essential component for the common goal. Predictive mechanisms in auditory processing can be examined by using brain signals for prediction violations like the mismatch negativity (Fujioka et al., 2004; Kujala et al., 2007). The mismatch negativity (MMN) occurs in response to a deviation in a musical sequence that the subject listens to. An enlargement of the MMN to rhythmic and melodic deviations within a musical sequence is seen in musicians (Fujioka et al., 2004; Vuust et al., 2005), and can be observed already after 2 weeks of musical training in previously untrained subjects (Lappe et al., 2008, 2011). Source analyses revealed that the increased MMN component is generated in overlapping, but partially different brain networks depending on whether a melodic or rhythmic deviant occurred. Whereas melodic deviations are processed in a temporo-frontal network, rhythmic deviations activate neurons along the temporo-parietal pathway. (Lappe et al., 2013a,b, 2016). This is consistent with the dual pathway model of auditory processing (Belin and Zatorre, 2000; Arnott et al., 2004; Rauschecker and Scott, 2009; Bizley and Cohen, 2013).

During duet performance, a fundamental difference between the own and the partner material is that the former is played, and hence predicted based on one's own motor performance, while the latter is available only from listening to the auditory signal provided by the partner's performance. In previous studies on cortical plasticity after short-term musical training we found that only playing, but not mere listening, led to an increased mismatch negativity to the trained material (Lappe et al., 2008, 2011). The specific situation in ensemble play, however, might require predictive representations of the partner's material too, and should, thereby, lead also to plasticity effects for that material. In the present study, we were therefore interested in whether duet training induces enhancement in the musical MMN also for the partner material, and whether the sources of the mismatch detection were similar or different for deviations in one's own and the partner's material. In duet training, it is an important question whether only own errors in the own material are relevant for one's own learning, or whether the errors of the partner are equally important. Melodic and rhythmic errors of a musical duet partner have as much consequences as one's own errors on the musical performance, and the duet partners' mistakes presumably influence the learning process of the other musician. Consequently, when both errors are equally important and electrophysiologically similarly represented in the brain, the musically elicited MMN should increase after a joint musical training for a tone deviation within the own and the partner's musical part.

Error attribution, however, is also important in a duet performance (Loehr et al., 2013). Although a partner's error has similar consequences for the performance, pianists strategies for error avoidance and error management are presumably different when the error was committed by oneself as compared to the partner. A simultaneous training of two participants in a duet situation allows to differentiate between own and partner errors. We, therefore, also looked for a neural signature of the distinction between deviances in the own and the partner material.

We trained non-musician subjects in a piano-duet task and measured mismatch components elicited by musical deviations within the pianist's own and the partner's part before and after training. In a subsequent beamformer source analysis we investigated the brain areas contributing to the generation of the mismatch negativity and the distinction of deviations within subjects' own and partner material.

## Methods

### Subjects

Sixteen non-musician subjects (9 females), aged between 19 and 34 years, took part in the experiment. They were recruited from the subject pool of the Institute of Biomagnetism and Biosignalanalysis in Münster and via newspaper advertisements, and were payed 9 Euros per hour for their participation. Participants had, in addition to their compulsory school lessons, little or no formal musical training (range of 0–5 years with a mean of 0.9 years, and a standard deviation of 1.6 years). None of them had received private piano lessons. All participants were right handed as assessed by the Edinburgh Handedness Inventory (Oldfield, 1971) and none had a history of ontologically or neurologically related disorders. No subject expressed impaired motor skills and all were able to perform the task motorically. Pure tone audiometry comprising a frequency range of 125–8,000 Hz was used to confirm that all participants had normal audiological status. All experimental procedures were in conformance with the Declaration of Helsinki. Subjects were fully informed about the nature of the study and all of them gave written informed consent to take part in the study.

### Procedure

The experiment consisted of three phases. In the first and third phase, we recorded MEG responses to deviants in a short section of a piano exercise while subjects listened to these stimuli. In the second phase, participants were trained in pairs to play a duet piano exercise that contained the MEG stimulus as an excerpt. We will first describe the training procedure and then the MEG procedures.

### Training procedure

**Figure 1:**
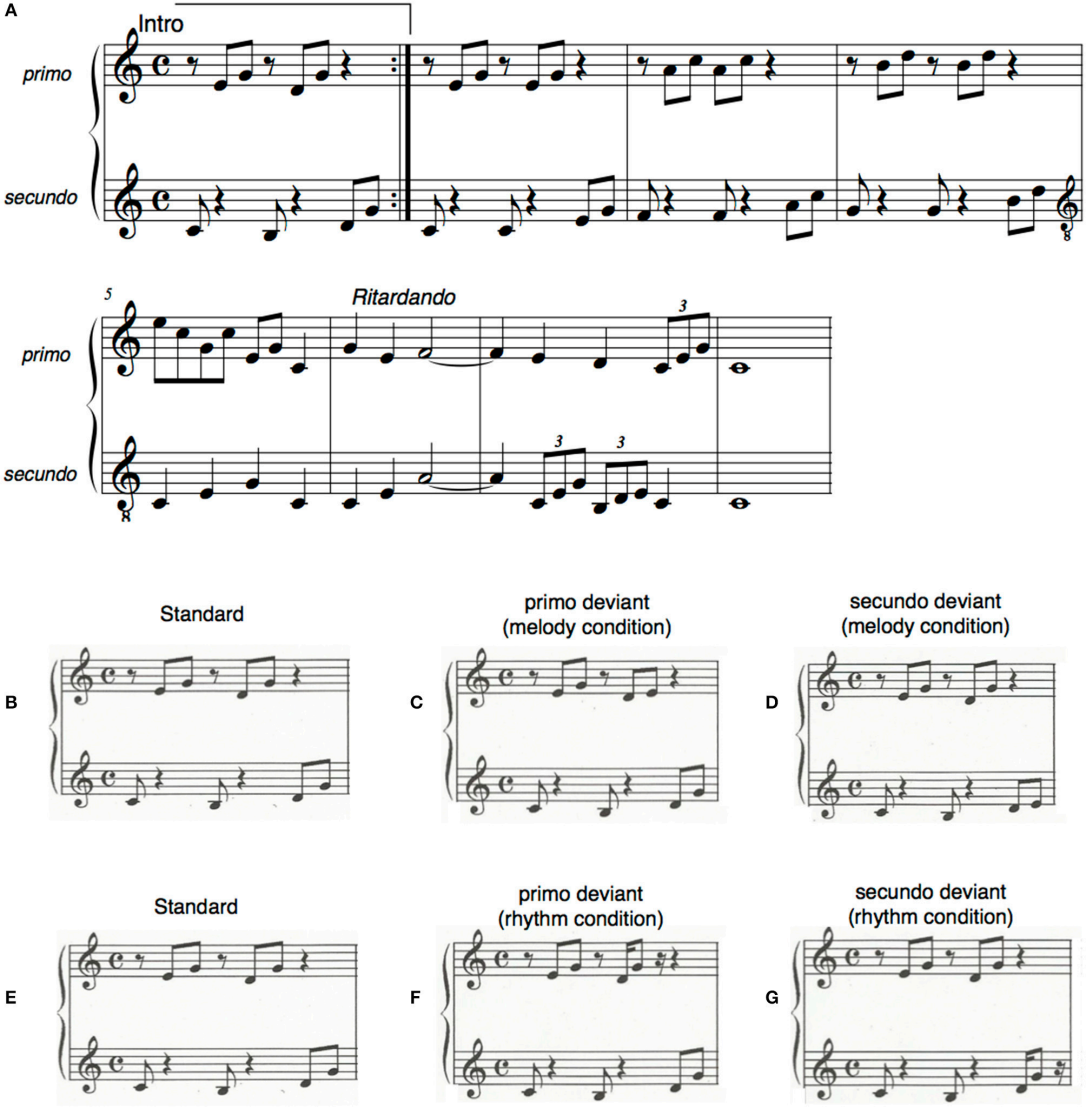
**(A)** The piano exercise subjects learned to play during the training sessions. The upper line was played by the primo, the lower line by the secundo part. **(B–G)** The stimuli that were used for the MMN measurement before and after the piano-duet training. The standard constitutes the intro of the exercise **(B,E)**. On deviant trials in the melody condition the sixth and eighth tones **(C,D)**, that were either part of the primo or the secundo material, were lowered by a minor third. In the rhythm condition the same notes were presented 70 ms earlier **(F,G)**.

The training exercise was a piano piece composed by the first author for the purpose of this study. It was based on the C-major chord progression in which the primo and secundo parts had the same number of notes (Figure 1A). In the first half of the piece the harmonies of C-major (tonic), F-major (subdominant), and G-major (dominant) were played in broken chords as a rising sequence. The notes of this section were played by the primo and secundo parts in an alternating fashion. In the second section of the exercise the melody went down and primo and secundo played, in contrast to the first part, simultaneously.

Subjects were grouped in pairs and were randomly assigned to either play the primo or the secundo part of the piano exercise. Training sessions were scheduled for each pair separately on 8 days over the course of 3 weeks. On average, training sessions were 1.5 days apart. Each session lasted 30 min. To facilitate training, a template was used where the piano keys were depicted and on which the finger placements were marked. At the beginning of the first training session an instructor played the piano exercise to give the participants an impression of how it should sound. Afterwards participants were familiarized with the template. The template was henceforth provided in all training sessions. Subjects were instructed to play from the beginning through the whole exercise. A single digital piano (Yamaha CLP) was used for both players and the training sessions were recorded via MIDI interface. At the beginning and end of the first training sessions an instructor listened to the performance of the piano duet and assisted when necessary. Apart from that, subjects were encouraged to work together on solutions for upcoming difficulties and mistakes during the musical training exercises. Training progress was monitored by measuring how often the intro part of the piano piece (the sequence used in the MEG) was played correctly during each half hour training session.

### MMN stimuli

For the pre- and post-training MMN measurement in the MEG the intro of the piano exercise was used (Figures 1B,E). The intro was composed of the tonic and dominant in broken chords in C-Major. The notes in this intro were equally distributed between the primo and the secundo parts: The first tone [C (251.63 Hz)] was played by the secundo part, the second and third tones [E (329.63 Hz), G (392.00 Hz)] were played by the primo part, the forth tone [B (246.94 Hz)] by the secundo, the fifth and sixth tones [D (293.66 Hz), G (392.00 Hz)] by the primo, and the seventh and eighth tones [D (293.66 Hz), G (392.00 Hz)] again by the secundo part.

The realistic piano tones for the stimuli in the MEG were generated by means of GarageBand (Apple Inc.) which is a digital audio workstation with an integrated on-screen virtual keyboard. The duration of each tone in the stimulus sequence was 300 ms resulting in a stimulus length of 2,400 ms overall. Successive sequences were separated by a silent interval of 500–700 ms. The musical sequences were presented in two runs using an oddball paradigm comprising 400 trials each (240 standard and 160 deviants).

To generate a mismatch negativity (MMN) to melodic changes within the primo and secundo musical material, 80 deviant sequences were altered in the first run by lowering the sixth tone, which was part of the primo training material, by a minor third (Figure 1C). In the remaining 80 deviant sequences of that run the eighth tone was lowered by a minor third (Figure 1D). This tone was part of the secundo training material. Melodic mismatch components were thereby built on physically identical intervals. Melodic deviants of the primo and the secundo parts were presented intermixed within the first run. Similarly, to generate a mismatch negativity (MMN) to a rhythmic change, the sixth (primo part) and eighth tones (secundo part) were presented 70 ms earlier (Figures 1F,G). Rhythmic deviants of the primo and secundo parts were presented intermixed within the second run of the MEG measurement. Rhythmically elicited MMNs of the primo and secundo part were, as in the melody condition, built on physically identical intervals.

### Data acquisition

The MEG recordings were performed in a magnetically and acoustically shielded room. Subjects were seated in an upright position and instructed not to move during the measurements. Their head positions were checked at the beginning and end of each recording block by means of three localization coils fixed to the nasion and the entrances of both ear canals. Subjects were asked to stay in a relaxed waking state during the measurement, and not to pay attention to the sound stimuli. To control for confounding changes in attention and vigilance, subjects watched a soundless movie of their choice (Fujioka et al., 2006; Boh et al., 2011; Okamoto and Kakigi, 2014). Alertness and compliance were verified by video monitoring. Magnetic field responses were recorded with a 275-channel whole-head magnetometer system (OMEGA 275; CTF Systems). MEG signals were low-pass filtered at 150 Hz and sampled at a rate of 600 Hz. The duration of a recording epoch was 2.6 s including 0.2 s pre stimulus interval, respectively. The data recording was synchronized to the stimulus presentation in each trial. The total recording time was 40 min.

### MEG data analysis

Epochs contaminated by muscle or eye blink artifacts containing field amplitudes larger than 2.8 pT in any of the 275 channels were automatically rejected from the averaging procedure. A 30 Hz low-pass filter was applied to the averaged field waveforms, and a baseline correction was performed based on the 100 ms time interval previous to the onset of the piano sequences. Data analysis was performed using the FieldTrip software (Oostenveld et al., 2011). For the analysis in sensor space, root mean square (RMS) values were calculated over the whole head for each subject for the averaged datasets of the standard and the deviant stimuli separately for the primo and the secundo parts. RMS values were then averaged over subjects for the standards and deviants of the own material (including the primo or secundo part), and the standards and deviants of the partner's material (including primo or secundo part). Subsequently, own and partner material was contrasted by subtracting deviant and standard waveforms of the own material and deviant and standard waveforms of the partner material. For the RMS analysis, a 2 × 2 within subject ANOVA on individual RMS values with factors pre/post-training and own/partner was calculated using the peak value of each individual subject within a time window of 90–300 ms after the deviant tone onset. A beamformer source analysis in the time domain (Linearly Constrained Minimum Variance; LCMV) was applied to determine the neural sources for error attribution. In beamforming, a spatial filter is constructed to estimate the contribution of a given source without imposing constraints on the source solution by determining the number and positions of the ECDs in advance (Hillebrand et al., 2005; Steinsträter et al., 2010).

T1-weighted anatomical MR images were acquired from each participant using a three Tesla Scanner (Gyroscan Intera T30, Philips, Amsterdam, Netherlands), and Turbo Field acquisition was applied to collect 400 contiguous T1- weighted 0.5 mm thick slices in the sagittal plane. Small containers filled with Gadolinium (to be visible in the MRI) were used for co-registration with the MEG measurements to mark the positions of the fiducial points. A realistically shaped head model implemented in the FieldTrip software was fitted to each individual participants' structural MRI and served as volume conductor model (Nolte, 2003). Segmentation was performed with the FieldTrip software. The volumetric structure contained the information about the different tissue types of the anatomical MRI and a probabilistic value was assigned to each voxel indicating to which tissue it belonged.

Individual anatomical MR images were warped to a template MRI in MNI coordinates which was subdivided into a regular three dimensional grid with a spatial distance of 1 cm between grid points. The source strength for each grid point was computed for predefined time windows producing a 3D dimensional spatial distribution of the power of the neural generators of the mismatch components. The time range of beamformer analysis was based on the time course of the RMS values. Since the melodically elicited MMN peaked 50 ms later and was broader in shape than the rhythmically elicited MMN, a time window of 100–250 ms was chosen for the melody MMN and a 90–150 ms time window was chosen for the rhythm MMN. The difference in response latency between rhythmic and melodic deviants is consistent with earlier findings (Lappe et al., 2008, 2011, 2016). A common spatial filter for the standard, deviant/own and deviant/partner conditions was computed separately for the melody and the rhythm conditions and then applied to calculate the strength of the source power. Statistical nonparametric mapping of the source power values was performed with a cluster-based randomization test to control for multiple comparisons. Alpha level was set at 0.05.

## Results

**Figure 2:**
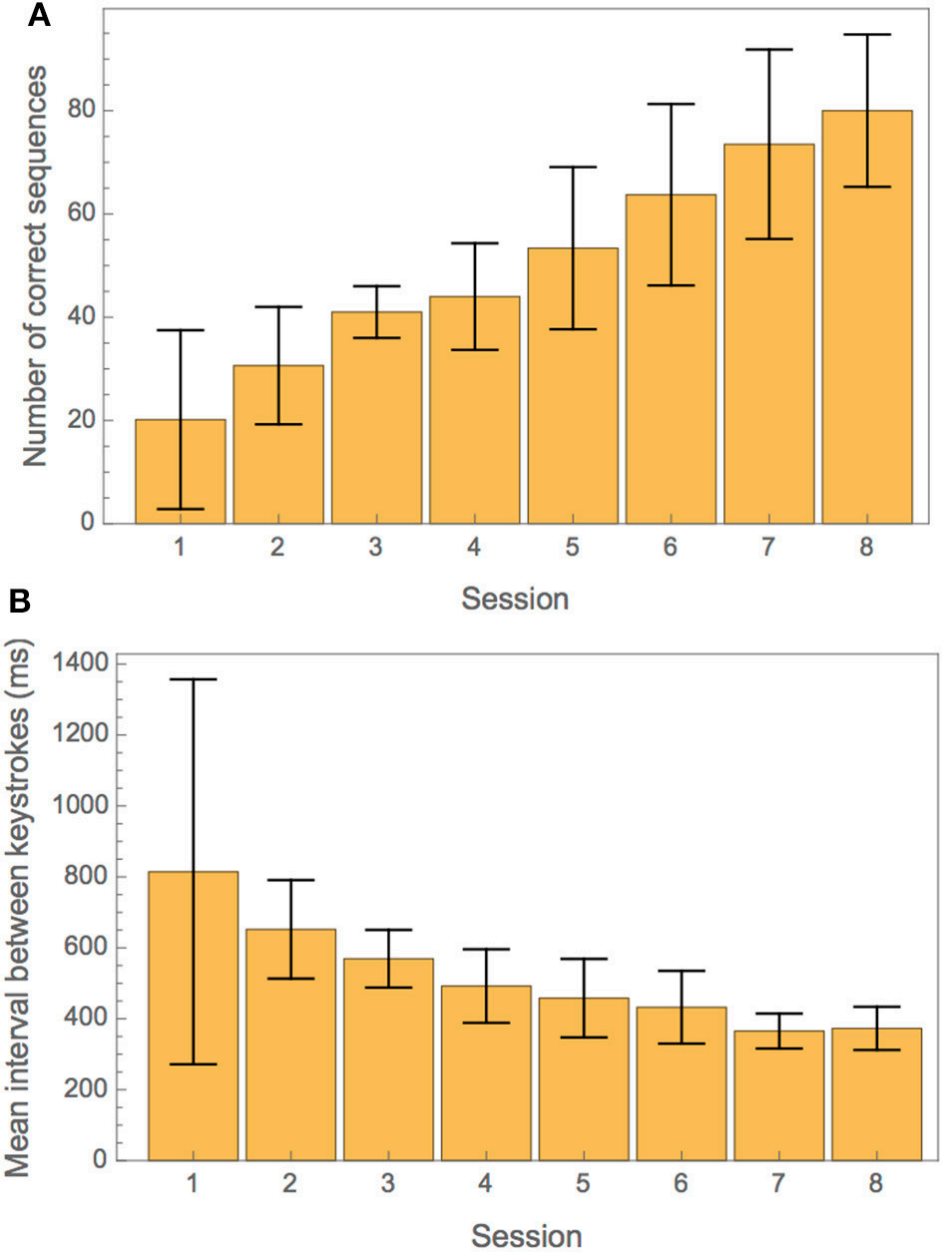
**(A)** Measures of training progress for correctness and tempo. **(A)** Average number of correct repetitions of the intro part of the exercise in each training session. **(B)** Mean interval between keystrokes in each training session.

Participants increased their ability to play the piano exercise successfully during training. This is illustrated in Figure 2. Figure 2A shows the average number of correct intro sequences played per training session. Figure 2B illustrates the progress in tempo over the course of all training sessions.

**Figure 3:**
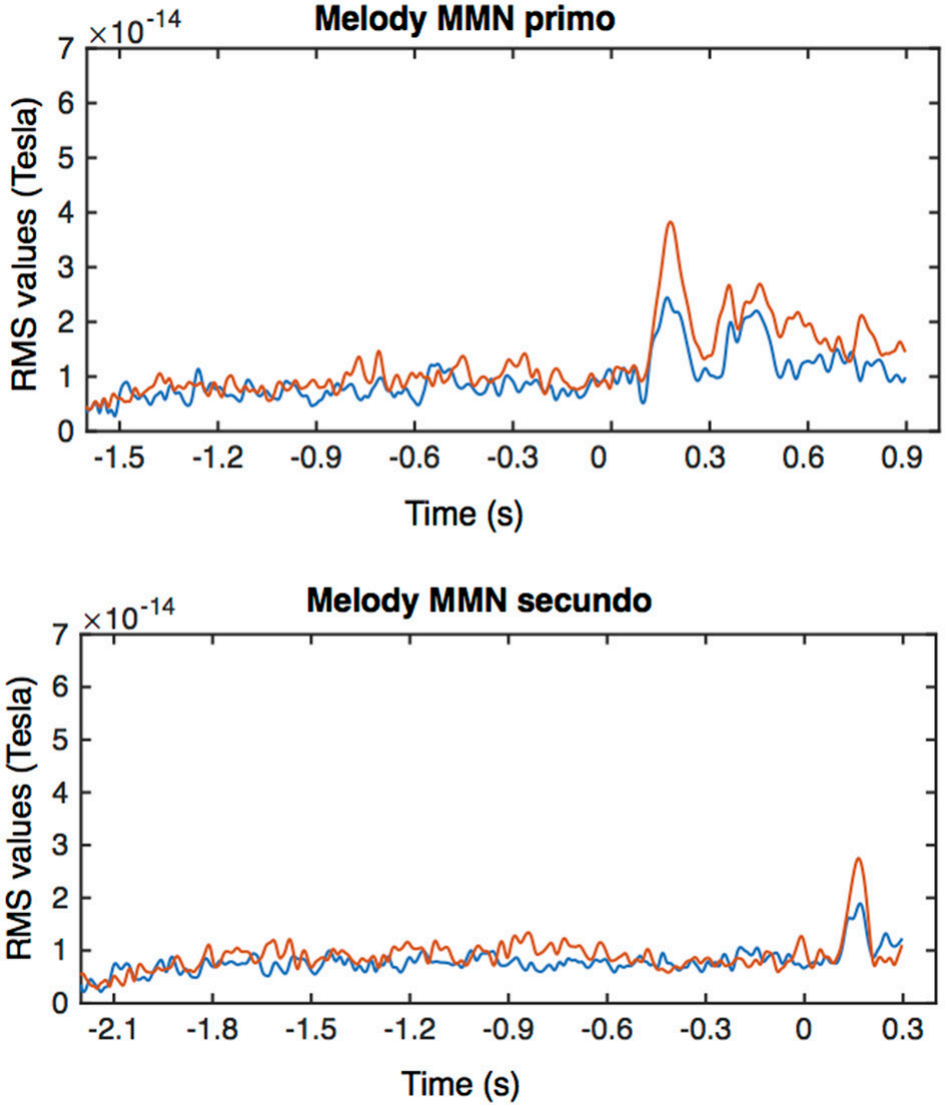
**Averaged RMS values for the MMN responses in the primo and secundo trials of the melody condition**. Blue lines indicate responses before training, red responses after training. Time is relative to the onset of the deviant tone, i.e., the 6th tone in the primo condition and the 8th tone in the secundo condition.

**Figure 4:**
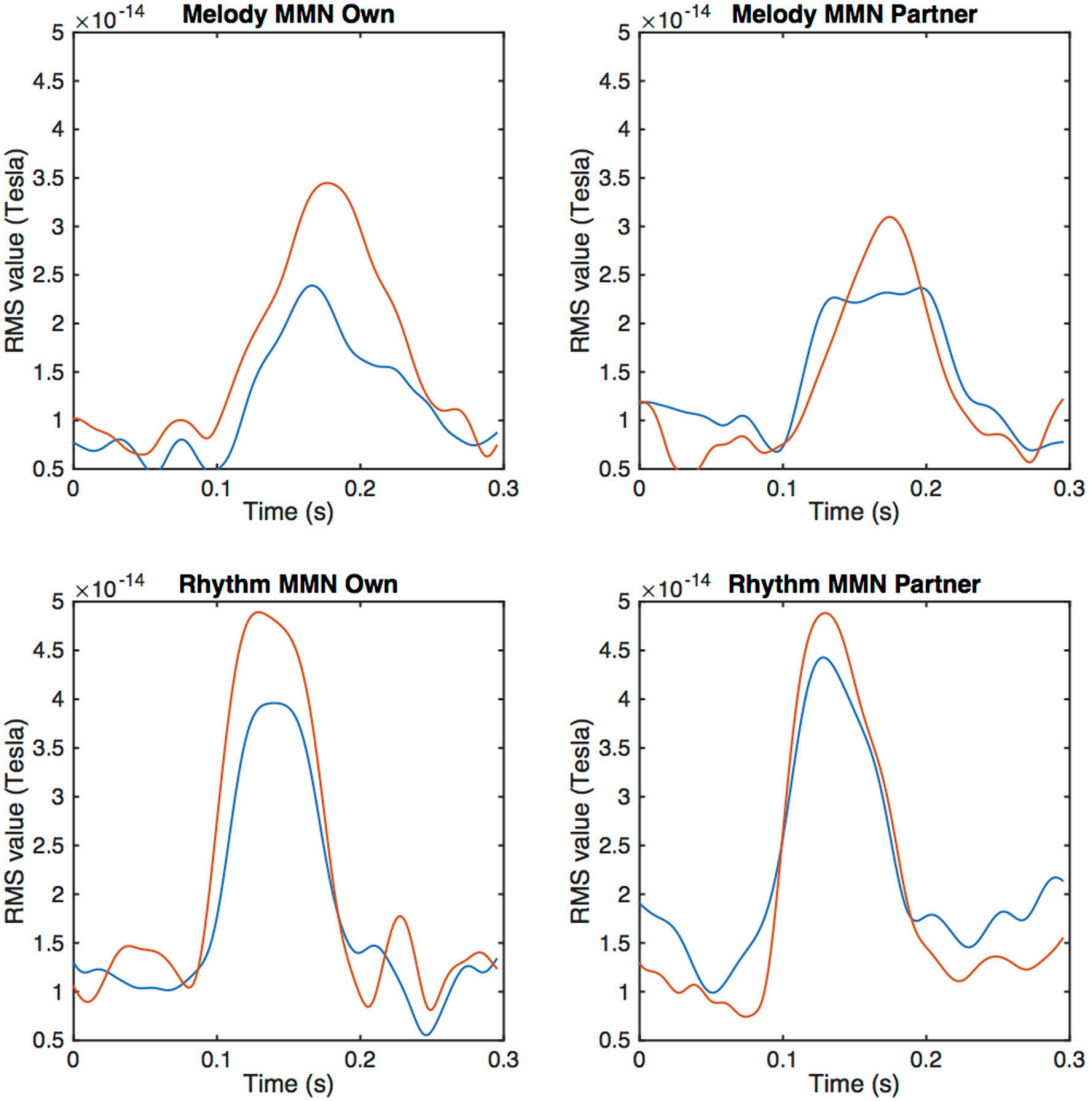
**Averaged peak amplitudes of RMS values for the MMN response after a melodic deviation contrasting own and partner material**. The upper row depicts the melody condition, the lower row the rhythm condition. Pre-training values are depicted by the blue, post-training data by the red curves.

From the MEG data, pronounced mismatch negativity components were noticeable in the RMS values after the tone deviation in the primo- and secundo parts before and after training. Figure 3 shows MMN RMS responses (deviant - standard) in the melody condition during the whole trial for the primo and secundo parts separately. Blue lines indicate responses before training, red lines indicate responses after training. In these figures, own and partner material are not yet separated. To study responses to own and partner deviations, we collected responses from both primo and secundo conditions and split these into own and partner material (Figure 4). Both, own and partner's material showed a clearly visible increase of the MMN after training in the melody as well as the rhythm condition. This observation was statistically supported in the melody condition by a within-subject ANOVA with factors own/partner and pretraining/posttraining, revealing a main effect of pretraining/posttraining [*F*_(1, 15)_ = 14.03; *p* = 0.04] and no interaction of own/partner X pretraining/posttraining. Similar results were found for the rhythm condition. A pretraining/posttraining main effect was also determined by a within-subject ANOVA with factors own/partner and pretraining/posttraining [*F*_(1, 15)_ = 7.19; *p* = 0.02]. No interaction effect was found between own/partner X pretraining/posttraining. We thus conclude that for both types of deviants the MMN increases after training, and, importantly, for one's own material as well as for the partner's material.

**Figure 5:**
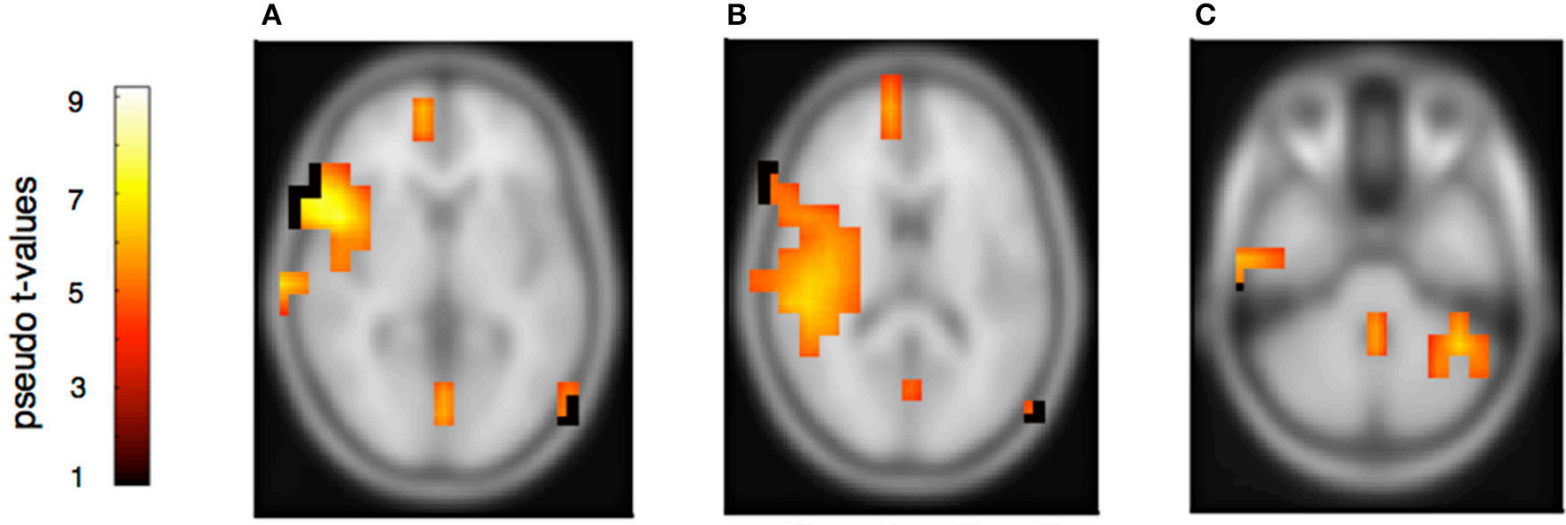
**Neural activation clusters after a melodic deviation within the own musical material**. Activation is found in right inferior frontal cortex **(A)** (*x* = 54, *y* = 14, *z* = 8), the right auditory cortex **(B)** (*x* = 54, *y* = −32, *z* = 16), the superior and medial frontal cortices **(A,B)** (*x* = 12, *y* = 62, *z* = 8) as well as the precuneus **(A,B)** (*x* = 16, *y* = −62, *z* = 28). Activation is also found in the left cerebellum contralateral to the right auditory and inferior frontal cortices **(C)** (*x* = −40, *y* = −50, *z* = −36).

**Figure 6:**
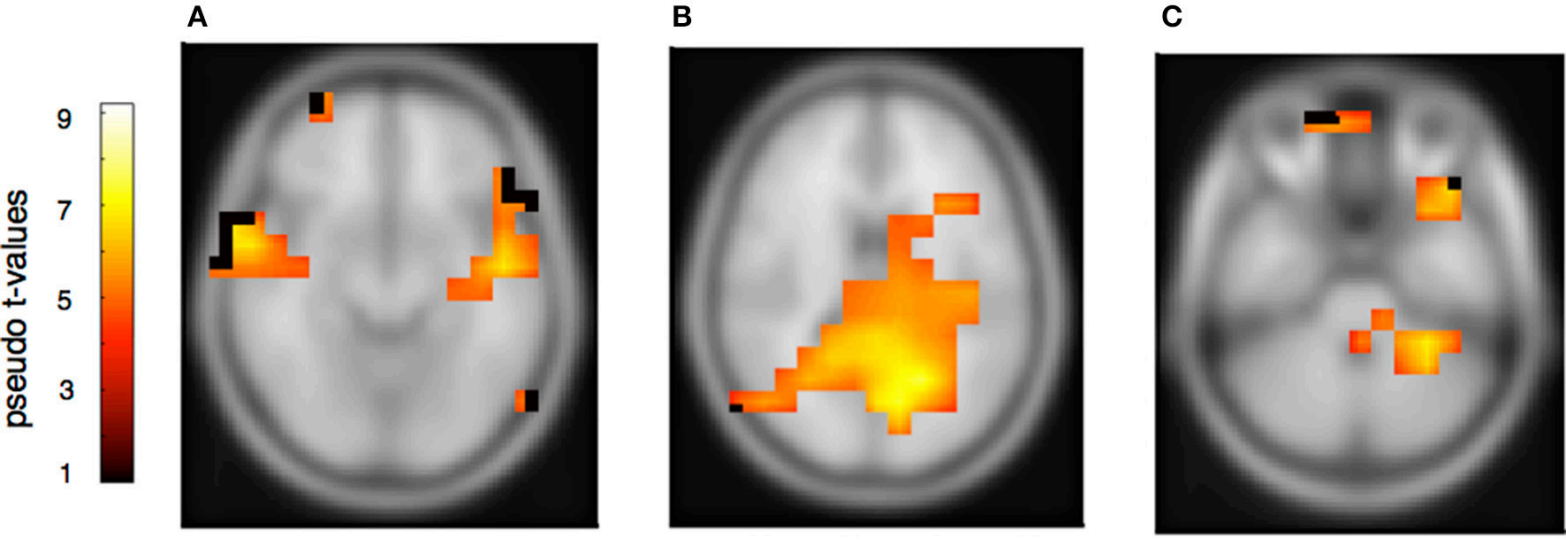
**Neural activation cluster after a melodic deviation within the partner's musical material**. Activation is found in the left (*x* = −50, *y* = −18, *z* = −8) and right anterior auditory cortices **(A)** (*x* = 52, *y* = −22, *z* = −12), and the precuneus **(B)** (*x* = 26, *y* = −52, *z* = 24). Activation is also found in the left cerebellum **(C)** (*x* = −30, *y* = −38, *z* = −40).

To determine the sources of the MMNs and a possible distinction between deviants in own and partners' parts, a beamformer analysis was conducted separately for the melodically and rhythmically elicited MMN and for the pianist's own and the partner's mismatch component. The melodically elicited MMN for pianists' own material revealed right sided neural activation in the anterior part of the auditory cortex (AC) and inferior frontal cortices (IFC) (Figure 5A, Table 1). Activation was also found in the precuneus and the medial frontal gyrus (Figures 5A,B) and, in addition, in the left cerebellum contralateral to the activation in the right auditory and inferior frontal areas (Figure 5C). Neural activation after a melodic deviation in the partner's material appeared less focussed, but also included activation of the AC in the left and right hemispheres (Figure 6A), the precuneus (Figure 6B), and the left cerebellum (Figure 6C). A direct comparison, however, of the pianist's own and partner's material did not reveal significant differences. We therefore conclude that a melodic deviation within a musical duet sequence elicits activation in the AC and IFC within one's own and the partner's material, representing a neural activation pattern that has been shown to also occur after a piano solo training (see Lappe et al., 2013a). In the piano duet situation of the present study, however, we find in addition neural activation in the precuneus, the medial frontal cortex as well as in the cerebellum in both players.

**Table 1:** **Observed peak voxel activation in MNI space**.

**Figure**	**Anatomical location**	**MNI coordinates**		
4	Right hemisphere	*x*	*y*	*z*
	Frontal superior medial	12	62	8
	Precuneus	16	−62	28
	Inferior frontal	54	14	8
	Superior temporal	54	−32	16
	Left hemisphere			
	Cerebellum	−40	−50	−36
5	Right hemisphere			
	Superior temporal	52	−22	−12
	Precuneus	26	−52	24
	Left hemisphere			
	Superior temporal	−50	−18	−8
	Inferior frontal	−38	16	26
	Precuneus	−20	−68	20
	Cerebellum	−30	−38	−40
6	Right hemisphere			
	Superior temporal	54	−36	16
	Cerebellum	22	−34	−30
	Left hemisphere			
	Inferior parietal	−44	−40	36
	Superior temporal	−50	−38	20
7	Right hemisphere			
	Temporal superior	60	−30	16
	Cingulum	14	16	36
	Left hemisphere			
	Frontal superior/medial	−20	56	14

**Figure 7:**
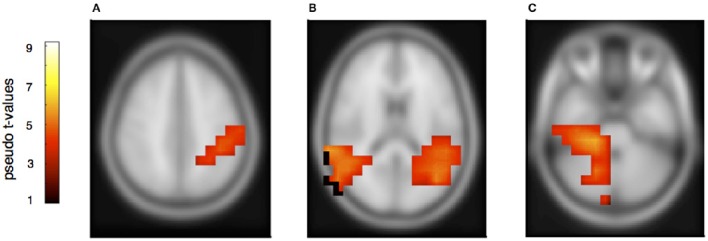
**Neural activation cluster after a rhythmic deviation within the own musical material**. Activation is found in the left inferior parietal area **(A)** (*x* = −44, *y* = −40, *z* = 36) and the left (*x* = −50, *y* = −38, *z* = 20) and right posterior auditory cortices **(B)** (*x* = 54, *y* = −36, *z* = 16). Activation in this rhythm condition is also found in the right cerebellum **(C)** (*x* = 22, *y* = −36, *z* = −30), contralateral to the left inferior parietal area.

**Figure 8:**
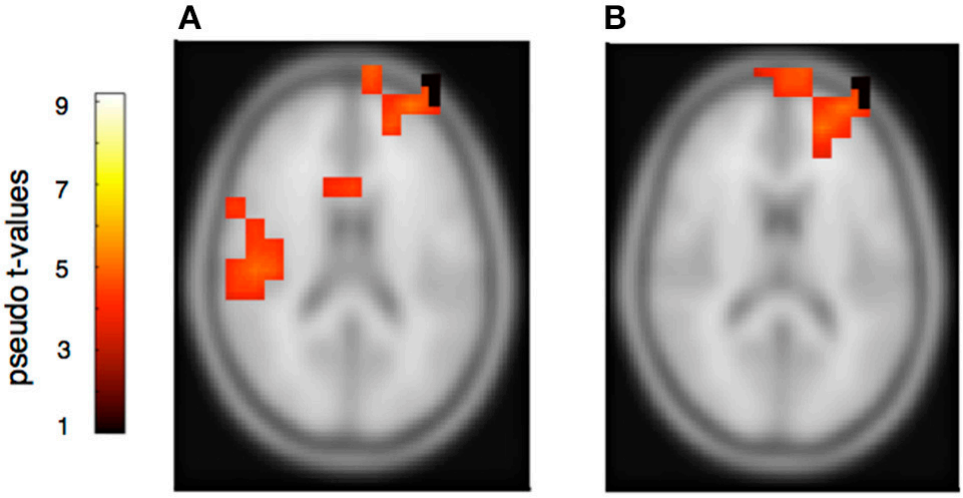
**Neural activation cluster after a rhythmic deviation within the partner's musical material**. Activation is found in the right auditory cortex **(A)** (*x* = 60, *y* = −30, *z* = 16), the cingulum **(A)** (*x* = 14, *y* = 16, *z* = 36) and the superior and medial frontal cortex **(A,B)** (*x* = −20, *y* = 56, *z* = 14).

The rhythmically elicited MMN in the pianist's own material concurred with activations in the left inferior parietal area (Figure 7A), the posterior part of the left and right auditory cortices (Figure 7B) and the right cerebellum contralateral to the left inferior parietal lobule (Figure 7C). For the partner material, MMN activation was elicited in the left auditory cortex, the cingulum and the left superior frontal and medial frontal cortex (Figures 8A,B). A direct comparison between own and partner material showed also no differences. We therefore conclude that a rhythmic deviation within a musical duet sequence elicits neural activation in the left and right AC and the left IPL within one's own and the partner's material, which is an activation pattern that has previously been shown to occur following a rhythmic deviation after a piano solo training (Lappe et al., 2013b). In the piano duet situation of this study, however, we find, in addition, neural activation in the frontal and medial frontal cortices as well as in the cerebellum in both players.

## Discussion

In the present study, non-musician subjects learned to play a musical exercise within a piano duet. Before and after training, we measured the musically elicited mismatch negativity that was generated by listening to deviations within the pianist's own or the partner's material. The MMN increased significantly after training for both parts. These plastic changes were observed both in the melodically and the rhythmically elicited MMN. This finding extends previous results showing training-induced plasticity effects only in musical material that was motorically trained but not after pure auditory training (Lappe et al., 2008, 2011; Maidhof et al., 2010). Specifically, in the study by Lappe et al. (2008) the MMN was larger after sensorimotor training compared to an auditory control group in which subjects listened to the same training material but did not play. The study by Maidhof et al. (2010) showed that the error related negativity (ERN) is larger when built up during musical performance compared to mere listening. The results of both studies suggest that expectancy is stronger when established during motor performance. The results of the present study support the hypothesis that in a duet situation, the partner's part, although only heard and not motorically learned, has a stronger significance compared to a mere listening situation. Duet partners need to work together to achieve a common, satisfying performance. The partner's part is therefore highly relevant for one's own progress and for the common goal, which therefore presumably leads to neural representations of the partner's musical part, too. Moreover, mistakes in the partner's part are highly important and discrimination accuracy, therefore, increases significantly also for the partner's musical material (Loehr et al., 2013).

Such a training-induced increase in the MMN to rhythmic or melodic deviation within a musical context points to an audio-motor coupling after sensorimotor training (Baumann et al., 2005; Bangert et al., 2006; Halwani et al., 2011; Jäncke, 2012; Mathias et al., 2015) leading to a better musical discrimination accuracy on the one hand and a better prediction ability about upcoming musical events on the other. It has been suggested that musical training establishes an internal forward model linking a musical tone with a specific motor movement resulting in strong associations between actions and their auditory consequences (Lee and Noppeney, 2011). Such an internal forward model would then predict the consequences of these actions by using efference copies of the motor command (Wolpert et al., 1995) helping the neural network to better detect auditory prediction violations, as manifested by the musical MMN. It has furthermore been suggested that, in a musical duet situation, each musician, since he or she learns to predict what the musical partner is doing, simulates the partner's musical part and establishes thereby an internal forward model for the partners material, too (Novembre and Keller, 2014; Hadley et al., 2015). Our results confirm this hypothesis. Duet pianists might have learned to form expectations not only by associating their own actions, but also by associating their partner's actions with specific auditory outcomes.

The mismatch negativity components generated for own and partner material were based on physically identical stimuli, thereby enabling a direct comparability of the mismatch enhancement after training. The associated motor command during training and the internal model of the outcome, however, were different since the tones had to be played at different time points in the training material.

Errors are important for learning. Brain activation related to errors in piano playing can be seen in components of electroencephalographic (EEG) data even before the onset of motor activity (Maidhof et al., 2009; Ruiz et al., 2009). The error-related negativity (ERN) is stronger during performance than during listening (Maidhof et al., 2010). Likewise, the mismatch negativity becomes stronger after training (Lappe et al., 2008, 2011). Larger error related ERF components following an error during or after a musical performance are presumably an indication that these errors are perceived by the musician as being highly relevant and important. In duet training, it is an essential question whether only own errors in the own material are relevant for one's own learning or whether the errors of the partner are equally important. Our results suggest that the latter is the case. A common performance is only possible when both players are able to accomplish their parts, and own and partner mistakes have equal consequences for the common goal. The increase of the MMN component for melodic and rhythmic deviations in the partner's part, as it was shown in our study, is therefore evident and plausible.

However, to learn from errors and to improve duet performance, players have to distinguish own errors from errors of the partner, attributing errors in relation to agency. Therefore, although the error related and the mismatch negativity components share similar neural representations, the requirement to attribute an error to oneself or to the partner has to be reflected somewhere in the neural network. Previous fMRI and ERP studies have mainly shown similar responses to one's own and to other's errors (de Brujin and Rhein, 2012; de Bruijn et al., 2012; Loehr et al., 2013). Loehr et al., for example, found similar feedback-related negativities for altered auditory stimuli during a piano duet performance no matter whether they occurred within the own or the partner's part. However, a larger P300 was seen within the own compared to the partner's material, suggesting that a differentiation of own and partners' action is processed at later times. Our beamformer results of own and partners' mismatch responses indicate also that the same networks are involved for own and partners' tone deviations since a direct subtraction of own and partner MMNs did not yield significant results. We therefore concentrate on those activation patterns that are similar for own and partner material. We find comparable neural activation for own and partner material in the melody condition, revealing neural activity in the auditory cortex in combination with inferior frontal cortex. This activation pattern has been shown in previous auditory deviance detection studies, especially after short- and long- term musical training (Lappe et al., 2013a,b, 2016) indicating that melodic deviations are processed in temporal-frontal networks. In addition, however, strong activation was found in the present duet study in the precuneus and the right superior medial frontal cortex. These brain areas have been implied in previous studies in the distinction between own and partners' actions (Frith and Frith, 1999; Amodio and Frith, 2006; Cavanna and Trimble, 2006; Isoda and Noritake, 2013). Hence, our data show that a neural signature of the necessity to differentiate between own and partner errors is already apparent at the early timing of the mismatch negativity component.

A further interesting point concerns the involvement of the cerebellum. The cerebellum is considered difficult to detect with MEG, since it is positioned rather distant from the sensors. Several recent MEG studies, however, have shown activation in cerebellar networks (Krause et al., 2010; Wibral et al., 2010; Fujioka et al., 2014; Lu et al., 2014). In the present study, cerebellar activation is consistently observed in almost all conditions and is clearly contralateral to the cortical activity, which is consistent with the opposing organization between cerebellar cortex and neocortex. Therefore, we are confident that the cerebellar activation in our data is of relevance. In fact, the cerebellum is considered an important part of the forward model of many motor behaviors (Wolpert et al., 1998). We therefore suggest that its involvement in the present study was built during the sensorimotor training process. However, since the mismatch measurements themselves do not involve motor behavior, our results suggest that the cerebellum contributes to prediction not only of motor actions but also of sensory sequences, consistent with recent findings from sensory and motor timing studies (Kotz et al., 2014).

This is also seen in the activation pattern after a rhythmic deviation in the own material. It revealed an involvement of the left and right auditory cortices, the left inferior parietal lobule, and the right cerebellum, contralateral to the left inferior parietal activity. This finding is again consistent with the cerebellum being part of the forward model built during the sensorimotor training. The fact that we found activation for the melody deviation in the left cerebellar hemisphere and for the rhythmic deviation in the right cerebellar hemisphere is especially interesting since it supports the general assumption that melodic sequences are processed more strongly in the right neocortical hemisphere whereas rhythm is stronger processed in the left neocortical hemisphere (Zatorre and Belin, 2001).

An involvement of the motor system in predicting musical sequences in the mismatch paradigm is sometimes, particularly in musicians, also shown by activation of cortical motor or premotor areas (Lappe et al., 2016). This was not the case in the present study. Perhaps a longer training is necessary to observe motor activation during the mismatch response using MEG and beamformer analysis. It is also possible that motor activation becomes visible in later components, such as the N200 (Mathias et al., 2015) that we could not analyze in our data set.

In summary, our results provide a view on the neural underpinnings of musical ensemble performance that suggests a common representation of the musical piece, acquired during joined training and established from predictive processes of sensorimotor performance together with an involvement of structures that differentiate the sources of any deviations.

## Author contributions

CL designed and conducted the experiment, analyzed the data and wrote the manuscript. SB performed the training. ML analyzed the data and wrote the manuscript. CP read and revised manuscript.

## Funding

The present work has been funded by the German Research Foundation DFG LA 3080/1-1.

### Conflict of interest statement

The authors declare that the research was conducted in the absence of any commercial or financial relationships that could be construed as a potential conflict of interest. The reviewer BM and handling Editor declared their shared affiliation, and the handling Editor states that the process nevertheless met the standards of a fair and objective review.
